# Effect of hypoxia on muscle activation at equivalent absolute and relative intensity during incremental and constant load exercise to task failure

**DOI:** 10.1113/EP093320

**Published:** 2026-01-06

**Authors:** Dania Ibrahim, Youmna Elsayed Hassanein, Nathan E. Townsend

**Affiliations:** ^1^ College of Health and Life Sciences Hamad Bin Khalifa University Doha Qatar

**Keywords:** electromyography, fatigue, hypoxia, incremental exercise, muscle activation

## Abstract

We examined the impact of moderate hypoxia (HYPO) on muscle activation during incremental exercise matched for both absolute and equivalent relative intensity. Fifteen active subjects (10 males, 5 females) completed two ramp incremental test and two step tests in normoxia (NORM; $F_{iO_{2}}$ = 0.209) and HYPO ($F_{iO_{2}}$ ≈ 0.135) in counterbalanced order. The respiratory compensation point (RCP) determined from ramp testing was used to normalize relative intensity during step testing, which included a final stage to task failure (TF) above RCP. Electromyography (EMG) was recorded for rectus femoris (RF), vastus lateralis (VL) and vastus medialis (VM), and normalized to a pre‐test maximal sprint effort. Linear mixed modelling was used to examine fixed effects of condition (NORM, HYPO) and intensity (absolute, relative) on EMG activity. During the ramp test, HYPO significantly reduced ${\dot{V}}_{O_{2}\mathrm{peak}}$ (∼13%), PPO (∼15%), and power at RCP (∼16%). EMG breakpoints occurred at lower absolute intensity in HYPO for RF and VL. When matched for relative intensity, muscle activity was lower in HYPO for VM and VL, but not RF. EMG activity at TF revealed a similar pattern whereby a strong association to absolute power was present regardless of test protocol or $F_{iO_{2}}$. These results suggest that altered relative metabolic stress has a negligible impact on muscle activation at work rates below the RCP. For exercise in the severe domain our data aligns with the theory that muscle activation is not critically regulated to a given level at TF, but appears to be task‐specific and independent of oxygen availability.

## Abstract (highlights)

- **What is the central question of this study?**
  Does exercise in hypoxia (HYPO) modulate muscle activation during steady state and non‐steady state exercise?
- **What is the main finding and its importance?**
  When performed at the same absolute work rate in moderate HYPO, electromyographic activity of quadriceps remained unchanged, whereas it was lower when relative metabolic stress was normalized. Muscle activation at task failure was strongly associated with power output regardless of $F_{iO_{2}}$. Moderate HYPO has little impact on muscle activation during cycling in the moderate and heavy intensity domains.

## 1 INTRODUCTION

Voluntary muscle contraction is precisely controlled by the central nervous system (CNS) to exert the force required to meet a given task. The appropriate level of muscle activation is met via a combination of motor unit (MU) recruitment and rate coding (Contessa et al., 2016), which together compose the surface electromyography (EMG) signal. Performance fatigability, defined as ‘an acute decline in motor performance caused by an exercise‐induced reduction in force or power of the involved muscles’ (Hunter, 2018), appears to play a role in modulating muscle activation whereby a combination of a gradual increase in MU firing rates with a decrease in recruitment threshold act synergistically to compensate for declining force of fatiguing MUs (Contessa & De Luca, 2013; Contessa et al., 2018). However, since the intensity of exercise affects both the force requirement and the underlying mechanisms that contribute to peripheral muscle fatigue, controlling for one variable, for example, power output (PO) while manipulating the other, requires an approach which dissociates the two. One such approach is exercise in acute hypoxia (HYPO), which increases the relative intensity at a given PO due to a decrease in maximal oxygen uptake (Wehrlin & Hallén, 2006) and estimates of the maximal metabolic steady state (MMSS) such as critical power (Dekerle et al., 2012; Shearman et al., 2016; Townsend et al., 2017), and the respiratory compensation point (RCP; Azevedo et al., 2020; Marwood et al., 2025).

During incremental exercise the relationship between recruitment order and MU size leads to emergence of a nonlinear EMG ‘breakpoint’ which occurs at intensity similar to or slightly greater than the RCP (Iannetta et al., 2017; Racinais et al., 2014; Scheuermann et al., 2002). Whether the EMG breakpoint is mechanistically related to MMSS, CP or RCP remains equivocal in humans; however, there is robust evidence in the rat that recruitment of type IIx muscle fibres is linked to critical speed (Copp et al., 2010). More broadly, the MMSS is recognised as an important ‘fatigue threshold’ above which the development of peripheral muscle fatigue continues unabated until task failure (TF) is attained (Poole et al., 2016). In numerous studies involving single‐joint isometric exercise, EMG activity gradually increases, which is interpreted as recruitment of additional MUs to compensate for the progression of muscle fatigue (Contessa et al., 2016, 2018; Katayama et al., 2007; Lai et al., 2021). Similarly, during larger muscle mass exercise (i.e., two‐legged cycling) at constant work rate (CWR) in the severe domain, EMG activity also increases progressively over time (Endo et al., 2007; Vanhatalo et al., 2011). These results are consistent with a mathematical model of the MU control scheme, which indicates that both the force requirement and metabolic cost of the task play a synergistic role (Lai et al., 2018).

During sustained CWR exercise below the MMSS, though, the capacity to attain an oxidative steady state is associated with ‘metabolic stability’ within contracting muscle, but the transition to this state is not instantaneous and is influenced by the primary component of oxygen uptake (${\dot{V}}_{O_{2}}$) kinetics (Grassi et al., 2011). Slower ${\dot{V}}_{O_{2}}$ kinetics invoke a greater O_2_ deficit, which in turn compromises metabolic stability and leads to a shift towards greater anaerobic energy contribution (Goulding et al., 2021). Arterial hypoxaemia is known to slow ${\dot{V}}_{O_{2}}$ uptake kinetics (Hughson & Kowalchuk, 1995) and can thus dissociate the normal relationship between external work rate and contribution of aerobic versus anaerobic energy contribution (Calbet et al., 2003). Exercise in HYPO is also typically associated with a shift towards greater carbohydrate utilization, even for intensities below the RCP (Lundby & Van Hall, 2002). Thus, it is generally accepted that exercise in HYPO at equivalent absolute intensity leads to a greater reliance on anaerobic pathways (Scott et al., 2016).

Consequently, numerous studies have reported accelerated fatigability during exercise in HYPO. For isolated quadriceps exercise, the rate of peripheral muscle fatigue development (estimated via twitch interpolation) is exacerbated in HYPO (Katayama et al., 2007; Ruggiero et al., 2022). For high intensity cycling exercise, the time to exhaustion (TTE) at a given PO was reduced in a HYPO in a dose–response manner (Romer et al., 2007). Conversely, when participants were allowed to self‐pace during a 5 km cycling time trial, they adopted a lower PO and took longer to complete the trial in HYPO (Amann et al., 2006). Interestingly in this study, EMG activity was lower in HYPO despite a similar magnitude of peripheral muscle fatigue, which illustrates the importance of absolute work rate when interpreting changes in EMG activity attributable to muscle fatigue. While the participants in this study reduced the workload via self‐pacing, it is also possible to normalize for relative intensity by anchoring the prescribed work rate to an estimate of MMSS.

Few studies have attempted to examine muscle activation using an experimental design that matches for both relative and absolute intensity, though. In one study, Torres‐Peralta et al. (2014) found greater EMG activity during incremental exercise in HYPO matched for absolute intensity; however, the intensity at which the EMG breakpoint occurred was not taken into account. Only one study has investigated the EMG breakpoint during incremental exercise in HYPO (Osawa et al. 2011), which occurred at a lower PO and ${\dot{V}}_{O_{2}}$ compared with normoxia (NORM). Both studies (Osawa et al., 2011; Torres‐Peralta et al., 2014) involved exercise in severe HYPO ($F_{iO_{2}}$ = 0.108–0.12) and moreover, the incremental test protocol used by Torres‐Peralta et al. (2014) used 2 min step durations only, and hence it is unclear whether EMG measurements were taken under steady state conditions, nor which stages were in the heavy versus severe intensity domains. Therefore, the aim of the present study was to examine the effect of moderate HYPO ($F_{iO_{2}}$ = 0.135) on muscle activation during incremental exercise matched for both absolute and equivalent relative intensity. A secondary aim was to examine muscle activation at TF during both ramp incremental and CWR exercise to exhaustion in the severe domain.

## 2 METHODS

### 2.1 Ethical approval

The experimental protocol for this study was approved by the Institutional Review Board of Hamad Bin Khalifa University (approval number: HBKU‐IRB‐2024‐33). All participants provided written informed consent after comprehensively elucidating the study's aims, methodologies, possible risks and advantages. Ethical considerations conformed to the most recent iteration of the *Declaration of Helsinki*, safeguarding participants' rights and welfare.

### 2.2 Experimental design

#### 2.2.1 Subjects

Fifteen healthy, physically active adults (10 males and 5 females) were recruited to participate in this study (mean ± SD age 25.2 ± 2.6 years, height 172.5 ± 7.8 cm, weight 68.4 ± 10.1 kg, ${\dot{V}}_{O_{2}\max}$ 45.5 ± 6 mL kg^−1^ min^−1^). Inclusion criteria required participants to be within an age range of 18–40 years, body mass index (BMI) of 18.5–24.9 kg m^−2^, non‐smokers, and to meet standard physical activity guidelines, that is, 150 min moderate to vigorous physical activity per week with cycling experience. Exclusion criteria included the presence of cardiovascular, pulmonary, neuromuscular or metabolic disease, a recent history of any medical event, including injury, surgery or illness, that occurred during the past 6–8 weeks, and no travel to altitudes >1500 m in the previous 3 months. All participants completed the Physical Activity Readiness Scale (PAR‐Q) prior to commencement in the study (Warburton et al., 2011).

#### 2.2.2 Study design

This study is a counterbalanced, placebo‐controlled crossover design. All subjects attended the temperature and humidity‐controlled laboratory (21 ± 0.5°C; 50 ± 5% RH) on four separate occasions, each visit separated by at least 48 h to minimize the effects of fatigue, and a maximum of 7 days. During the first two visits, participants completed a ramp incremental test either in NORM ($F_{iO_{2}}$ = 0.209) or normobaric HYPO ($F_{iO_{2}}$ ≈ 0.135) in counterbalanced order. During visits 3 and 4, the participants completed a step incremental test in NORM or HYPO (in the same order ramp testing), where each step work rate was normalized for equivalent relative intensity between conditions. Normobaric HYPO was generated using a nitrogen dilution system (AltiTrainer, Nyon, Switzerland). The hypoxic stimulus is equivalent to ∼3300 m, which remains within a ‘moderate’ HYPO/altitude region that limits the direct effect of cerebral deoxygenation on supraspinal fatigue (Millet et al., 2012) and a decrease in work above CP (Townsend et al., 2017). Single‐blinding was implemented by activating the hypoxic generator for all test sessions, but for NORM the gas blender intake was room air. Participants were instructed to maintain their usual exercise regime during the entire experiment period, refrain from high‐intensity exercise on the day before, and complete no prior exercise on the day of testing. As part of a broader study, participants complied with a dietary control protocol which involved a minimum of 6 h no food or caffeinated beverages prior to testing for afternoon sessions or an overnight fast of 8–10 h for morning test sessions.

### 2.3 Experimental protocols

#### 2.3.1 Baseline peak sprint power

All exercise testing was performed on an electromagnetically braked cycle ergometer (Lode Excalibur Sport, Groningen, The Netherlands). Prior to ramp and step tests, a baseline EMG normalization procedure was completed, consisting of 2 min of cycling at 20 W, followed by one brief (10 s) effort at 2 W kg^−1^ and 4.5 W kg^−1^. Then, two maximal effort isokinetic sprints lasting 6 s, were performed under each distinct experimental condition, that is, NORM and HYPO, thereby facilitating condition‐specific normalization of the EMG signals. Each work bout was followed by 2 min of recovery at 20 W. All isokinetic mode maximal sprint efforts were fixed at a cadence of 80 rpm. In the event of a poor maximal effort or instances where the cadence was not within ±3 rpm of 80, a third sprint was completed, and the best two were used for further analysis. The EMG recorded during the maximal dynamic efforts served as the 100% value against which all other EMG measures were normalized (further details below).

#### 2.3.2 Ramp incremental test

The ramp rate for the incremental test was determined by first estimating each participant's ${\dot{V}}_{O_{2}\max}$ via a non‐exercise questionnaire validated for a similar subject population as the present cohort (Nes et al., 2011). Then, a standard metabolic cost equation was used to estimate the PO required to elicit ${\dot{V}}_{O_{2}\max}$ and the ramp rate was solved (to the nearest 5 W min^−1^) to reach this intensity in 10 min. In practice, this produced only three ramp rates of 20, 25 and 30 W min^−1^, which thus represent a pseudo ‘iso‐time’ protocol. The incremental test commenced with a 5 min baseline at 20 W, and immediately switched to the ramp phase thereafter and continued until volitional exhaustion. Participants were instructed to cycle at 80 rpm throughout the test. TF, that is, test termination, was defined as the inability to maintain the cadence above 70 rpm for ∼5 s despite strong verbal encouragement. The same ramp test protocol was used for both NORM and HYPO conditions. The RCP was determined in both conditions (details below) and served as the ‘anchor’ point to calculate each workload performed in the step incremental test.

#### 2.3.3 Step incremental test

The step incremental test commenced with a 4 min baseline cycling at 20 W followed by six constant work rate (CWR) stages and one TTE stage. The highest CWR stage (i.e., step 6) was set at 80% of each participant's estimated PO at RCP. This PO was divided by six to create an equidistant step increment resulting in relative intensities for stages 1–6 as follows: 13%, 27%, 40%, 53%, 67% and 80% of RCP. Steps 1–3 were 4 min in duration, step 4 was 6 min, and steps 5 and 6 lasted 8 min. The purpose of increasing step duration with intensity was to minimize total test duration but ensure a true steady state was achieved within the heavy domain (Iannetta et al., 2020). The work rate for the final TTE stage was fixed at 40% of the difference between peak power output (PPO) and RCP. This intensity was selected based on pilot testing, which indicated a typical TTE in the range of 2–4 min and thus clearly within the severe intensity domain at an intensity that would elicit peak ${\dot{V}}_{O_{2}}$ at or near ${\dot{V}}_{O_{2}\max}$ obtained during ramp testing. Participants were again instructed to maintain a cadence of 80 rpm (for all stages) and during the TTE to continue cycling until volitional fatigue, defined as per the ramp test (i.e., inability to maintain 70 rpm).

### 2.4 Measurements and data processing

#### 2.4.1 Pulmonary gas exchange variables

Throughout all exercise testing, cardiorespiratory variables were continuously measured breath‐by‐breath using a metabolic cart (Cosmed Quark PFT, Rome, Italy). Before each test session, the metabolic cart gas analyser was calibrated with ambient room air and a commercially prepared gas mixture (O_2 _= 15.95%; CO_2_ = 4.00%). A 3 L syringe was used to calibrate the volume turbine across a range of different flow rates, and an automated sample line delay check procedure was performed. Breath‐by‐breath ${\dot{V}}_{O_{2}}$ data were initially filtered to remove aberrant breaths and outlier data points (>2 SD away from a local mean of nine consecutive breaths) and then all gas exchange variables plotted against metabolic rate (i.e., ${\dot{V}}_{O_{2}}$) on the *x*‐axis.

For ramp incremental testing, estimation of the gas exchange threshold (GET) and RCP was carried out by two expert reviewers using standard criteria as follows: GET, the first non‐linear increase in ${\dot{V}}_{E}/{\dot{V}}_{O_{2}}$ without a corresponding increase in ${\dot{V}}_{E}$/${\dot{V}}_{CO_{2}}$, accompanied by a flattening of the $P_{\mathrm{ETC}O_{2}}$ marking onset of isocapnic buffering; RCP, the second non‐linear increase in ${\dot{V}}_{E}/{\dot{V}}_{O_{2}}$ accompanied by a non‐linear increase in ${\dot{V}}_{E}/{\dot{V}}_{CO_{2}}$ and marked decline in $P_{\mathrm{ETC}O_{2}}$ (Beaver et al., 1986; Keir et al., 2015). The mean response time (expressed as a PO equivalent in watts) was estimated at 2/3 and 1.0× the ramp rate for GET and RCP, respectively (see Discussion for methodological considerations here). The highest 30 s moving average for ${\dot{V}}_{O_{2}}$ was defined as ${\dot{V}}_{O_{2}\mathrm{peak}}$. All cardiorespiratory variables are reported as a 20 s centre‐weighted average, coincident with the respective GET, RCP and ${\dot{V}}_{O_{2}\mathrm{peak}}$. For step incremental testing, all cardiorespiratory variables are reported as the average of the last 60 s of each stage below RCP and the final 20 s average immediately prior to TF in the TTE stage.

#### 2.4.2 Heart rate and rating of perceived exertion

Heart rate (HR) was measured using a Trigno Duo Sensor (ADInstruments, Oxford, UK) utilizing two chest leads and recorded by LabChart Pro software. Rating of perceived exertion (RPE) was recorded every 2 min during the ramp tests and within the last 30 s of each stage for the step test, using Borg's 1–10 category‐ratio scale (Borg, 1982).

#### 2.4.3 Electromyography data analysis

**FIGURE 1:**
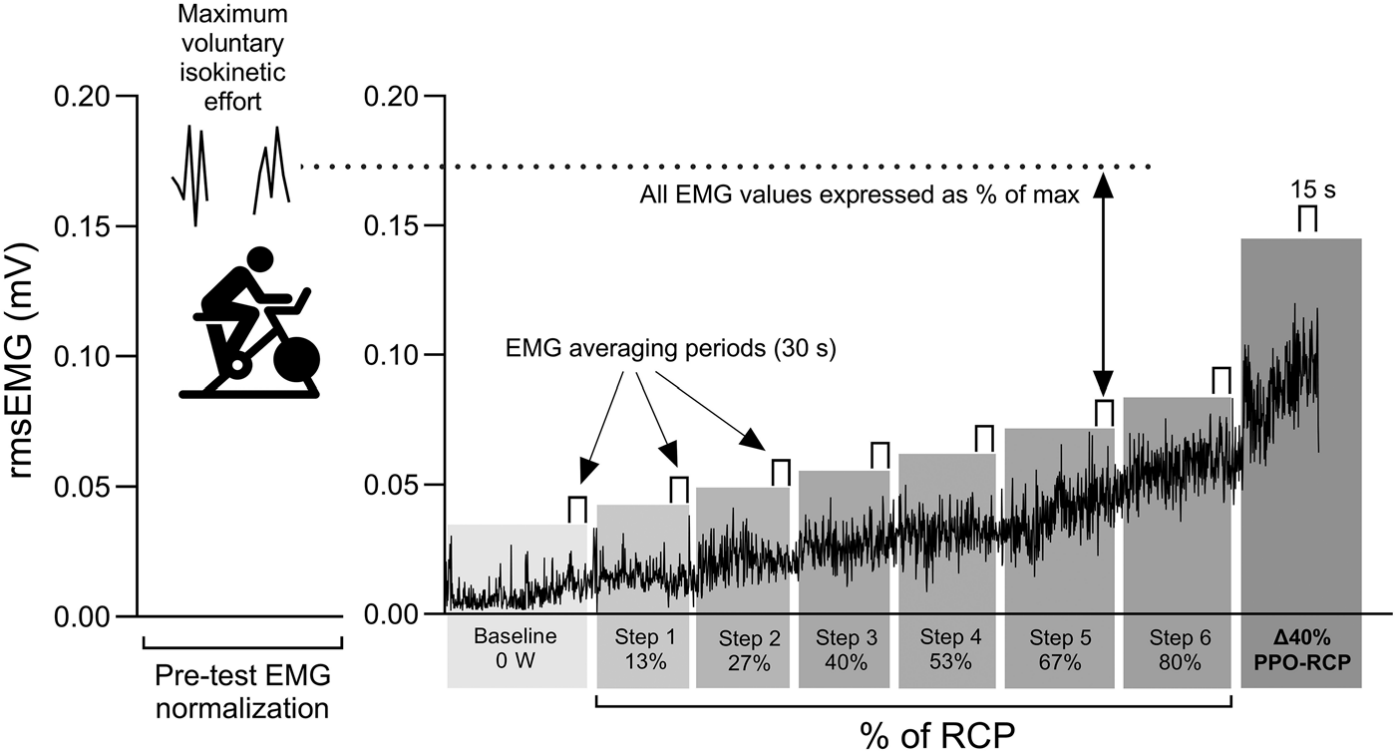
Example electromyography (EMG) data analysis for a typical subject to determine percentage muscle activation during the step test (with same analysis procedure used for ramp testing). Shaded grey bars refer to the intensity used for each step. The average of two maximal isokinetic efforts (6 s at 80 rpm) was taken as 100% rmsEMG against which all other measures were normalized. NB, the pre‐test EMG normalization was conducted in the same condition (i.e., normoxia [NORM] or hypoxia [HYPO]) as the following ramp or step tests.

Surface EMG of rectus femoris (RF), vastus medialis (VM) and vastus lateralis (VL) was measured continuously throughout all exercise testing using a wireless electrode system (Trigno Wireless System, Delsys, Boston, MA, USA) incorporating bipolar parallel‐bar surface electrodes linked to a multichannel data acquisition system (PowerLab 4/25T, ADInstruments). Before applying the sEMG sensors, each participant's skin was shaved and cleansed with alcohol wipes. The electrodes were positioned over each muscle belly following SENIAM guidelines, and fixed in place using an elastic bandage wrap. The raw sEMG signals were differentially amplified with a gain of 20 mV and common mode rejection of <80 dB. Data were recorded at a sample rate of 2000 Hz using LabChart Pro software (v8.1.2.8, ADInstruments) and a bandpass filter applied with a cut‐off frequency range of 20–450 Hz. The post‐acquisition signal was smoothed using a root mean square (RMS) envelope with a 200 ms moving window and zero overlap. An automatic moving baseline was selected, continuously detecting the baseline value between muscle contractions throughout the recording period. Muscle contraction onset and offset were defined as 10% of the peak height (above baseline) for each contraction and a minimum period of 500 ms between successive peaks (i.e., equivalent to a cadence of 120 rpm). The total area under the rmsEMG curve and above the baseline was computed for each revolution (units of V s). For the maximal isokinetic effort, the first three pedal revolutions coinciding with PPO were averaged, and this value was taken as the dynamic maximal activity. The rmsEMG activity for all pedal revolutions was averaged into 20 s bins for the ramp test. For the step test, the average over the final 30 s of the first six stages and the final 15 s before the limit of tolerance was reached in the TF stage were computed. Thereafter, all rmsEMG values in units of V s were expressed as a percentage of the pre‐test dynamic maximum (Figure 1) for breakpoint and statistical analysis.

The EMG breakpoint, expressed as PO (W), was determined using dual‐segment least squares linear regression (GraphPad Prism version 10.4.1, GraphPad Software, Boston, MA, USA). All data were first screened manually, and only tests in which an increasing deflection could be visually identified were used in statistical analysis. Of the 13 participants with successful CPET test data, two were omitted for RF and VM, and four were omitted for VL due to technical issues or poor signal quality. These omissions were not within the same participants. The first 2 min of the ramp phase were ignored to avoid detecting the first EMG breakpoint as recommended (Iannetta et al., 2017).

#### 2.4.4 Statistical analysis

Initially, Q–Q plots and the Shapiro–Wilk test were used to check the normality of all variables. Linear mixed modelling (LMM) was used to examine fixed effects of condition (NORM vs. HYPO) during both ramp and step incremental testing for all work rates up to the RCP, using a different model structure for analysis of either relative intensity or absolute intensity effects. In each case, the experimental condition (NORM, HYPO) was a fixed effect. When intensity was expressed in absolute units, the PO (W) was added as a covariate (ABS model), whereas when intensity was expressed in relative terms as a percentage of RCP, it was added as a second fixed effect (REL model). Individual participant intercept and slope were added as random effects initially, and then Akaike's information criterion (AIC) was assessed with or without the addition of individual slope as a random factor. The model with the lowest AIC was used unless there was a failure of convergence (or near convergence) warning, and in these cases individual slope was removed as a random factor. A LMM was also used to examine differences in EMG activity at TF, and in this case, condition (NORM vs. HYPO) and test type (ramp vs. step) were included as fixed effects. PO was independently examined as a covariate (fixed effects of conditions and test type were ignored). The restricted maximum likelihood method was used for all LMM analyses and performed using Jamovi version 2.3.2 (GAMLj3 module). Main effects and *post hoc* analysis results are reported as estimated marginal means (EMMs) with 95% confidence intervals in text and tables. The LMM statistics reported are *P*‐values, marginal *r^2^* denotes the model variance derived from fixed effects alone, and conditional *r^2^* encompasses variance from both fixed and random effects (Nakagawa & Schielzeth, 2013). Pearson's correlation was used to investigate the relationship between EMG and gas exchange breakpoints. Student's paired *t*‐test was used for variables with pairwise comparisons between HYPO and NORM only (e.g., EMG breakpoint and at TF). Cohen's *d* was used to denote effect sizes *post hoc* for pairwise comparisons, including *post hoc* analysis. Cohen's *d* effect sizes were interpreted as small (*d* = 0.2), medium (*d* = 0.5) and large (*d* = 0.8) as suggested by Cohen (1988). Statistically significant differences were defined as *P *< 0.05. All raw data are presented as means ± SD.

## 3 RESULTS

### 3.1 Ramp incremental test

#### 3.1.1 Physiological responses

The effects of HYPO on ramp incremental test variables are presented in Table 1. There were significant decreases in HYPO for ${\dot{V}}_{O_{2}\mathrm{peak}}$ (13%), PPO (15%), the PO at RCP (16%), and PO at GET (14%). The ${\dot{V}}_{O_{2}}$ at GET and RCP was also significantly reduced in HYPO. Conversely, there were no significant differences in ${\dot{V}}_{E}$ and HR at GET, RCP or ${\dot{V}}_{O_{2}\mathrm{peak}}$. Also, the percentage of ${\dot{V}}_{O_{2}\mathrm{peak}}$ at GET and RCP was not significantly different between NORM and HYPO.

**TABLE 1:** Physiological variables measured at gas exchange thresholds and maximal oxygen uptake during ramp incremental testing.

	Condition		*Post hoc* analysis	
Variable	NORM	HYPO	*P*	Cohen's *d*
**GET**				
Power output (W)	92 (33)	79 (20)	**0.05**	**0.36**
%${\dot{V}}_{O_{2}\max}$	58.9 (5.9)	56.9 (3.2)	0.25	0.31
${\dot{V}}_{O_{2}}$ (L min^−1^)	1.77 (0.38)	1.48 (0.21)	**0.02**	**0.70**
${\dot{V}}_{E}$ (L min^−1^)	39.9 (7.8)	40.5 (7.0)	1.00	0.04
HR (bpm)	130 (18)	135 (13)	0.38	0.57
**RCP**				
Power output (W)	153 (43)	128 (26)	**0.001**	**0.67**
% ${\dot{V}}_{O_{2}\max}$	79.8 (5.6)	78.7 (4.8)	0.52	0.17
${\dot{V}}_{O_{2}}$ (L min^−1^)	2.40 (0.50)	2.05 (26)	**0.006**	**0.85**
${\dot{V}}_{E}$ (L min^−1^)	66.8 (17.2)	65.1 (8.7)	1.00	0.10
HR (bpm)	158 (14)	156 (13)	0.93	0.26
${\dot{V}}_{O_{2}\max}$				
Peak power output (W)	255 (50)	217 (49)	**< 0.001**	**0.98**
${\dot{V}}_{O_{2}}$ (L min^−1^)	3.02 (0.61)	2.62 (0.41)	**0.003**	**0.96**
${\dot{V}}_{E}$ (L min^−1^)	115.9 (23.0)	110.6 (26)	0.47	0.32
HR (bpm)	176 (8)	170 (15)	0.29	0.62

Abbreviations: GET, gas exchange threshold; HR, heart rate; HYPO, hypoxia; NORM, normoxia; RCP, respiratory compensation point.

#### 3.1.2 Breakpoint analysis

**FIGURE 2:**
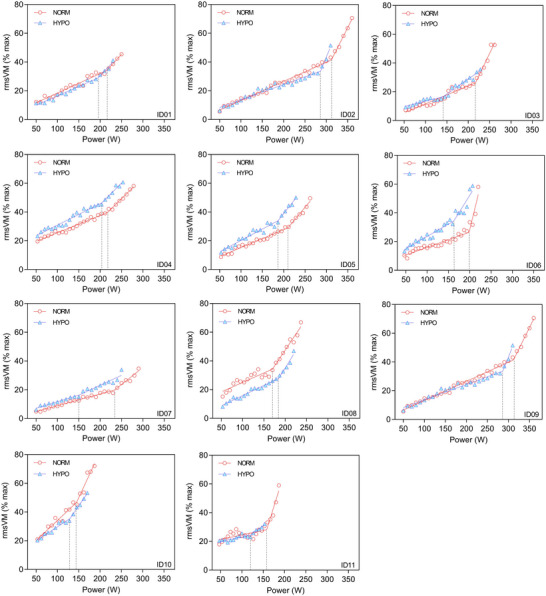
Electromyographic breakpoint analysis during incremental cycling under normoxia (NORM) and hypoxia (HYPO). Each panel shows data for an individual participant (*n* = 11 successful analyses) for vastus medialis (VM). Dashed vertical lines indicate the electromyography (EMG) breakpoints in each condition.

Figure 2 shows individual participant data for the EMG breakpoint analysis for VM, while Table 2 presents group mean data for RF, VL and VM. The PO at the EMG breakpoint was significantly reduced in HYPO for RF and VL, but not VM. There were no significant differences in rmsEMG at the EMG breakpoint in any muscle expressed as a percentage of dynamic maximal activity. In NORM, the PO at RCP was significantly correlated with PO at EMG breakpoint for RF (*P* = 0.0474; *r* = 0.61) and VM (*P* = 0.0482; *r* = 0.60), but not VL (*P* = 0.386; *r* = 0.33). In HYPO, there were no significant correlations between PO at RCP and the EMG breakpoint for RF (*P* = 0.498; *r* = 0.23), VM (*P* = 0.466; *r* = 0.26), or VL (*P* = 0.172; *r* = 0.47). In NORM, the mean PO at RCP was significantly lower than PO at EMG breakpoint for RF (*P* = 0.00207; *d* = 1.24) and VM (*P* = 0.00267; *d* = 1.30), but not VL (*P* = 0.0875; *d* = 0.65) (NB, mean data for PO shown in Tables 1 and 2). Similarly, in HYPO the mean PO at RCP was significantly lower than PO at EMG breakpoint for RF (*P* = 0.00427; *d* = 1.11) and VM (*P* = 0.00313; *d* = 1.26), but not VL (*P* = 0.0546; *d* = 0.71).

**TABLE 2:** Electromyography (EMG) breakpoint analysis for rectus femoris (RF), vastus medialis (VM), and vastus lateralis (VL) muscles.

	Condition		Paired *t*‐test	
Variable	NORM	HYPO	*P*	Cohen's *d*
**EMG breakpoint: power output (W)**				
Rectus femoris (*n* = 11)	204 (56)	184 (41)	**0.04**	**0.71**
Vastus medialis (*n* = 11)	198 (49)	184 (49)	0.36	0.29
Vastus lateralis (*n* = 9)	184 (30)	166 (33)	**0.049**	**0.77**
**EMG breakpoint: rmsEMG %max**				
Rectus femoris	25.5 (9.3)	23.8 (11.5)	0.52	0.20
Vastus medialis	32.4 (8.2)	32.9 (12.3)	0.87	0.05
Vastus lateralis	29.0 (11.2)	28.0 (13.2)	0.72	0.13

Abbreviations: HYPO, hypoxia; NORM, normoxia; rms, root mean square.

#### 3.1.3 Absolute intensity EMG analysis

**FIGURE 3:**
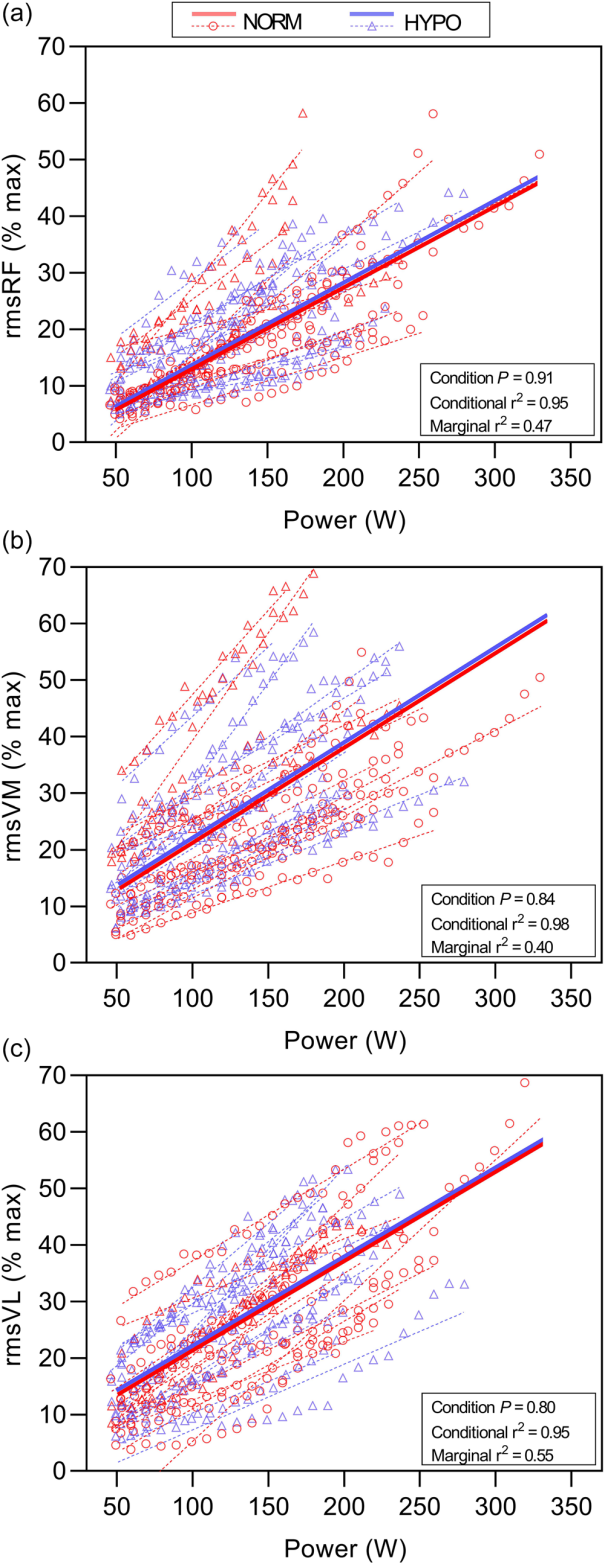
Muscle activity during the ramp incremental test expressed as rmsEMG percentage of pre‐test dynamic maximal values for rectus femoris RF (a), vastus medialis VM (b), and vastus lateralis VL (c). The 20 s bin average observed data is shown for normoxia (NORM) (red circles) and hypoxia (HYPO) (blue triangles) including individual linear regression slopes (dotted lines). The thick red (NORM) and blue (HYPO) straight lines denote the linear mixed model (LMM) predicted effects.

Figure 3 presents quadriceps muscle activation estimated by rmsEMG in NORM and HYPO conditions. When expressed as a function of absolute exercise intensity, that is, PO in Watts, there was no significant difference between NORM and HYPO for rmsRF% (*P* = 0.91), rmsVL% (*P* = 0.80), or rmsVM% (*P* = 0.84). In each case, there were large differences between the conditional and marginal *r* ^2^ values, which indicate a high proportion of the overall model variance is explained by random effects relative to the fixed effect (i.e., condition).

### 3.2 Step incremental test

#### 3.2.1 Physiological variables

When normalized for equivalent relative intensity during steady‐state exercise below RCP, there was no effect of HYPO on HR (EMMs NORM: 116 [104, 124] vs. HYPO: 120 (112, 127] bpm; *P* = 0.10) or ${\dot{V}}_{E}$ (EMMs NORM: 36.8 [33.4, 40.2] vs. HYPO: 36.1 [32.7, 39.5] L min^−1^; *P* = 0.57). In contrast, there was a significant effect of HYPO on ${\dot{V}}_{O_{2}}$ (EMMs NORM: 1.52 [1.39, 1.65] vs. HYPO: 1.34 [1.21, 1.47] L min^−1^; *P* < 0.001).

#### 3.2.2 Relative intensity EMG analysis

**FIGURE 4:**
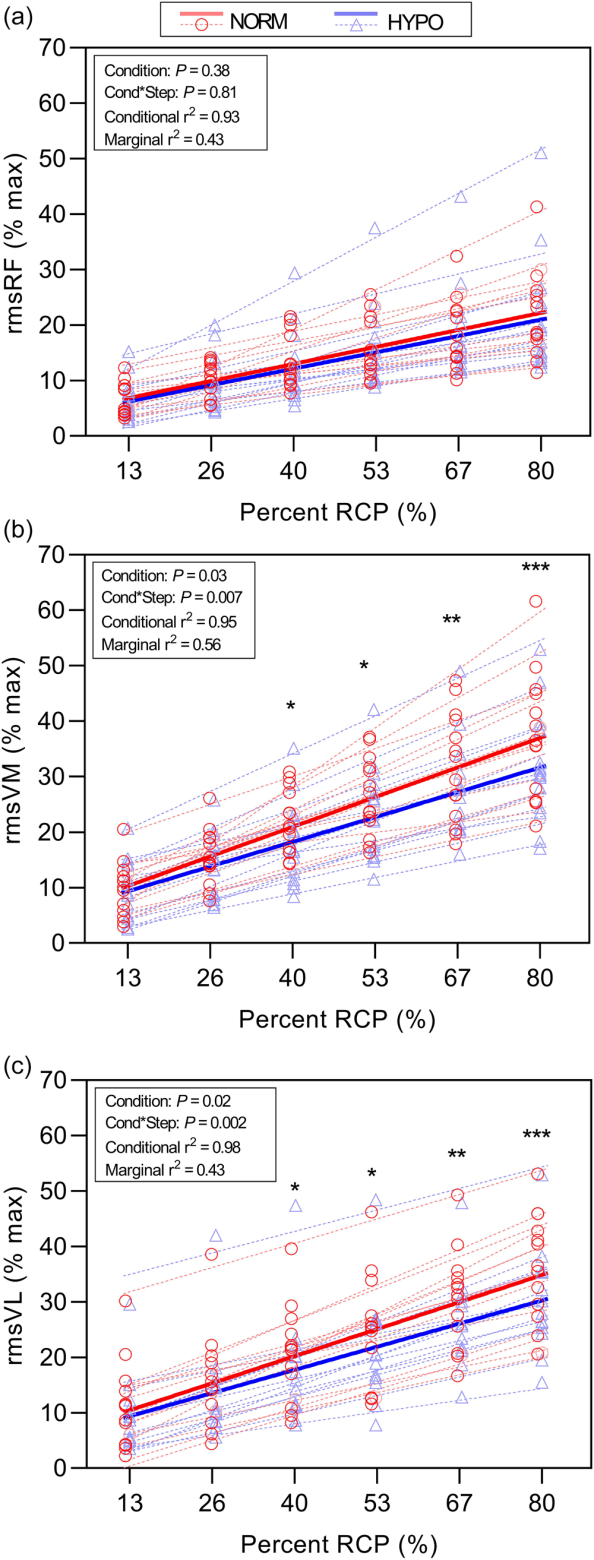
Muscle activity during the step incremental test expressed as rmsEMG percentage of pre‐test dynamic maximal values for rectus femoris RF (a), vastus medialis VM (b), and vastus lateralis VL (c). Observed data is shown for NORM (red circles) and HYPO (blue triangles) including individual linear regression slopes (dotted lines). The thick red normoxia (NORM) and blue hypoxia (HYPO) straight lines denote the linear mixed model (LMM) predicted effects. Asterisks denote significant *post hoc* differences (HYPO vs. NORM) at the intensity shown. *P < 0.05, **P < 0.01, ***P < 0.001.

Muscle activation for RF, VM and VL is shown in Figure 4. The effect of HYPO on rmsVM revealed a significant effect of power on rmsVM% (*P* = 0.03) and a significant interaction between condition and step (*P* = 0.007). The conditional *r^2^* for this model was 0.95, while the marginal *r^2^* was 0.56, indicating a similar proportion of variance explained by fixed and random effects. For rmsVL%, a significant effect of power (*P* = 0.02) was observed, along with a significant condition × step interaction (*P *= 0.002). The conditional *r^2^* = 0.98, while the marginal *r^2^* was 0.43. For rmsRF% there was no significant effect of power (*P* = 0.38) or condition × step interaction (*P* = 0.81). The model exhibited a conditional *r^2^* = 0.93 and marginal *r^2^* = 0.43, again indicating that a similar proportion of overall variance was attributed to fixed and random effects.

### 3.3 Time to exhaustion stage

#### 3.3.1 Physiological variables

Mean PO in NORM was 192 ± 41 W and 170 ± 34 W in HYPO. TTE was not significantly different (*P* = 0.164; *d* = 0.38) between NORM (230 ± 180 s) and HYPO (170 ± 146 s). Upon reaching TF in the TTE stage, peak ${\dot{V}}_{O_{2}}$ was 14% lower in HYPO (2.50 ± 0.46 L min^−1^) compared with NORM (2.91 ± 0.56 L min^−1^; *P* = 0.003; *d* = 1.03). There was no significant difference in peak ${\dot{V}}_{E}$ in NORM (99.5 ± 20.5 L min^−1^) versus HYPO (99.0 ± 17.7 L min^−1^; *P* = 0.88, *d* = 0.04), or peak HR in NORM (174 ± 6 bpm) versus HYPO (172 ± 8 bpm; *P* = 0.49, *d* = 0.21). When compared to peak measured values during the ramp test, there was no significant difference in ${\dot{V}}_{O_{2}\mathrm{peak}}$ at TF in either NORM (*P* = 0.25; *d* = 0.33) or HYPO (*P* = 0.33, *d* = 0.28). All participants reported a maximum rating of perceived exertion (RPE) (i.e., 10) at TF in both conditions.

#### 3.3.2 EMG analysis

**FIGURE 5:**
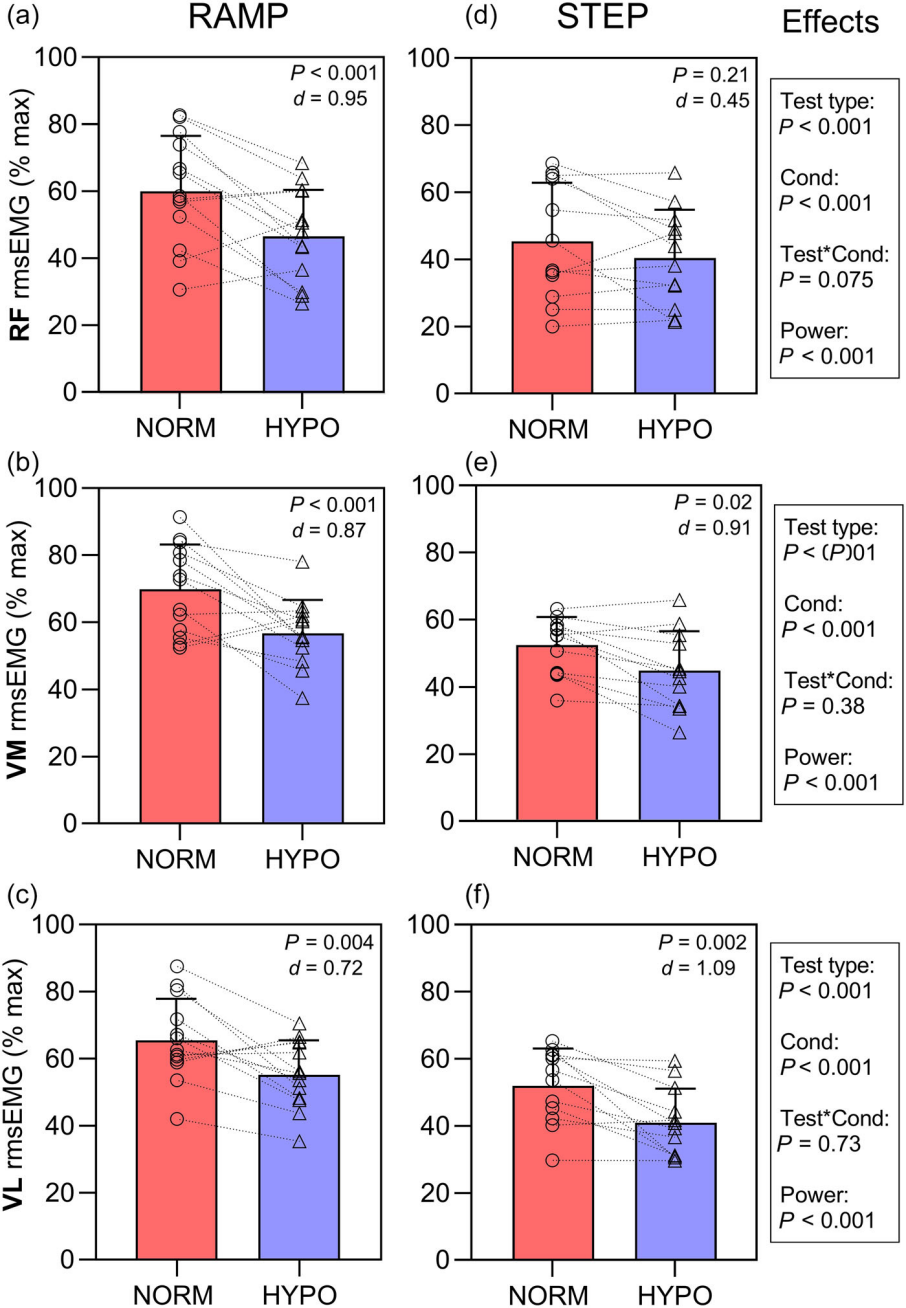
Muscle activation expressed as a percentage of the maximum of the pre‐test dynamic maximal contraction root mean square (rms) electromyography (EMG) for the RAMP test for rectus femoris RF (a), vastus medialis VM (b), and vastus lateralis VL (c) and for the STEP test for RF (d), VM (e), and VL (f). Displayed data are shown for NORM (red bar) and HYPO (blue bar). Effects statistics refer to results of linear mixed model (LMM) including power output as a covariate.

Figure 5 presents the mean (SD) and individual data for rmsEMG (%max) occurring at TF in both the ramp (a–c) and step (d–f) tests. Results of LMM revealed significant effects of condition (HYPO lower than NORM) and the test type (Step TTE stage lower than PPO in the ramp test) for each muscle (all *P* < 0.001). When condition and test type were removed as fixed effects from the model, there was a significant effect of power on rmsEMG at TF for all muscles (all *P* < 0.001).

## 4 DISCUSSION

The main findings of this study were that a HYPO‐induced increase in relative intensity did not modulate muscle activity during ramp incremental exercise at equivalent absolute work rates, whereas during CWR exercise normalized to the RCP, the EMG activity of VM and VL was lower in HYPO. Furthermore, a significant effect of PO on EMG activity was present at TF regardless of condition or test protocol. Lastly, the EMG breakpoint occurred at a lower absolute, but not relative intensity in RF and VL (but not VM) in HYPO. These findings do not support a clear modulating role for HYPO on muscle activation during cycling below the RCP either directly, or indirectly due to elevated relative intensity.

### 4.1 Effect of hypoxia on RCP and EMG breakpoints

During exercise in moderate HYPO, CP is reduced in an approximately linear manner with decreasing $P_{iO_{2}}$ (Townsend et al., 2017), and the magnitude of this decrease is similar to reductions in ${\dot{V}}_{O_{2}\max}$ (Wehrlin & Hallén, 2006). While controversy exists concerning the agreement and interchangeability between CP and RCP (Broxterman et al., 2018; Caen et al., 2022; Leo et al., 2017; Micheli et al., 2025), and also EMG breakpoints with either RCP or muscle oxygenation thresholds (Racinais et al., 2014), a physiological rationale has been suggested for a common underlying mechanism (Keir et al., 2024). In HYPO, there is evidence that RCP is diminished by a similar magnitude as CP (Azevedo et al., 2020; Marwood et al., 2025), and the NIRS‐derived muscle oxygenation breakpoint has been shown to decrease in moderate HYPO (Azevedo et al., 2020; Marwood et al., 2025). Also, a recent study confirmed that maximal lactate steady state (MLSS) is decreased in HYPO (Beever et al., 2024). In the present study, we observed a ∼15% decrease in RCP in HYPO compared to NORM, which is consistent with previous reports and expected for the magnitude of HYPO used (Azevedo et al., 2020; Girard et al., 2015; Marwood et al., 2025). We observed a significant, ∼10% decrease in the absolute PO where the EMG breakpoint occurred for RF and VL (but not VM), which is consistent with one other study that examined EMG activity in VL only (Osawa et al., 2011). When expressed as a percentage of pre‐test dynamic maximum, though, there was no change in HYPO for any muscle (Table 2). Thus, regardless of whether there is close agreement between different physiological breakpoints during incremental cycling, that is, RCP, NIRS and EMG, what does appear to be consistent is that each threshold decreases during exercise in HYPO, along with CP and MLSS determined from constant load test protocols. The precise mechanisms via which reduced convective O_2_ transport decreases the intensity where these physiological thresholds occur are not fully understood; however, a plausible explanation is offered by Goulding et al. (2021), based in part on predictions from in silico mathematical modelling (Korzeniewski & Rossiter, 2021). According to this theory, the shift from the heavy to severe intensity domain is coincident with a ‘critical [P_i_] threshold’ above which declining work efficiency initiates a positive feedback loop, leading to a continuous increase in non‐steady state oxidative metabolism until TF is reached. Since incremental ramp exercise represents a modality in which the O_2_ deficit continuously accumulates, in cases where the fundamental ${\dot{V}}_{O_{2}}$ uptake kinetics time constant is slower, for example in untrained or unhealthy populations compared to trained (Goulding et al., 2021), the rise in the O_2_ deficit occurs more rapidly, and therefore the ‘critical [P_i_] threshold’ is attained at a lower absolute work rate. Similarly, during exercise in HYPO, ${\dot{V}}_{O_{2}}$ uptake kinetics are typically slowed (Engelen et al., 1996; Hughson & Kowalchuk, 1995; Spencer et al., 2012), which could thus represent the key underlying mechanism that reduces the MMSS.

### 4.2 Matched absolute intensity

In the present study, the same ramp incremental protocol was used in both NORM and HYPO conditions. When expressed as a function of absolute PO in watts, we did not observe a change in the magnitude of EMG activity. This finding contrasted with our hypothesis that EMG activity would increase and differs marginally from the results of Torres‐Peralta et al. (2014) documenting heightened muscular activation during conditions of acute severe HYPO ($F_{iO_{2}}$ = 10%). This situation is classified within the severe hypoxic spectrum and signifies a markedly diminished proportion of $F_{iO_{2}}$ relative to that utilized in the current study. It remains unclear exactly how moderate HYPO could modulate muscle activation, since in vivo methods to interrogate the corticospinal pathway are technically challenging to implement during dynamic exercise such as running or cycling. Two hypothetical mechanisms are (1) HYPO could directly enhance MU recruitment and/or firing rate for a given force requirement, and (2) there could be an indirect influence in response to development of peripheral muscle fatigue. Evidence for the former mechanism is lacking, since most studies which added HYPO during isometric tasks failed to find an increase in corticospinal excitability using external stimulation techniques such as transcranial magnetic stimulation and/or peripheral nerve stimulation (Amann & Dempsey, 2008; Goodall et al., 2010; Jubeau et al., 2017). Also, during whole‐body exercise in severe HYPO ($F_{iO_{2}}$ ≈ 10.5%) a reduction in cortical voluntary activation and corticospinal excitability has been observed (Goodall et al., 2014). Therefore, it appears unlikely that moderate HYPO directly magnifies descending motor drive or excitability of the corticospinal tract.

Rather than HYPO per se acting directly to modulate muscle activation, peripheral muscle fatigue could impact MU recruitment and firing rate to maintain force output. Substantial evidence of this mechanism exists in studies involving single‐joint, isometric and dynamic exercises and high‐density EMG measurements (Contessa et al., 2016; Goodall et al., 2010; Lai et al., 2021). In particular, MU decomposition during fatiguing muscle contractions illustrates a gradual increase in firing rate of already recruited MUs combined with additional recruitment of new MUs, which together elicit higher EMG activity despite constant force output (Vanhatalo et al., 2011). However, we did not observe elevated EMG activity in HYPO in any muscle during the ramp incremental test. Note that we omitted data above the RCP from this analysis so that a decrease in the EMG breakpoint would not confound the results, whereas Torres‐Peralta et al. (2014) included data up to volitional exhaustion during incremental exercise, and reported an increase in EMG activity mainly at higher work rates. Similarly, during single‐leg knee extension in HYPO matched for absolute intensity, EMG activity was greater than in NORM, but the relative intensity was within the severe domain (Fulco et al., 1996). Together, these results suggest the magnitude of peripheral fatigue which occurs during incremental exercise below the RCP is insufficient to elicit an increase in EMG activity.

The magnitude of HYPO may also play a role since it has been reported that muscle activity increases during cycling when the $F_{iO_{2}}$ is below 0.12 (Amann et al., 2006; Taylor et al., 1997). In contrast, no change was observed in EMG of RF, VM and VL during cycling when $F_{iO_{2}}$ = 0.13 (Taylor & Bronks, 1995) or in VL EMG activity at $F_{iO_{2}}$ = 0.15 (Amann et al., 2006). Moreover, the magnitude of active muscle mass engaged during exercise in HYPO impacts the efficiency of pulmonary gas exchange, and thus, for a given $F_{iO_{2}}$, there is a greater arterial O_2_ desaturation during whole‐body large muscle exercise (Calbet & Lundby, 2009). Aligning with this, Scott et al. (2018) found elevated EMG activation while performing back squats in moderate HYPO ($F_{iO_{2}}$ = 16%). It should be noted, though, the relative intensity during these resistance exercise tasks is much higher than RCP. Hence, the level of active muscle mass engaged may act synergistically with the magnitude of HYPO, that is, a *greater* reduction in $P_{iO_{2}}$ may be required the smaller the muscle mass engaged, and also the relative intensity of exercise, that is, a *lesser* reduction in $P_{iO_{2}}$, the higher the intensity, to accelerate peripheral fatigue sufficiently such that an alteration to MU firing rate behaviour occurs.

Lastly, when several muscle groups and muscles within a group are engaged simultaneously, force distribution can vary dynamically (Contessa & Luca, 2013; Kouzaki et al., 2002). For instance, the knee extensors use ‘force‐sharing’ to sustain PO by activating or coactivating certain muscles more, and others less (Hering et al., 2023; Hug et al., 2015). Such a mechanism, especially at lower relative intensities, may attenuate the development of fatigue within one specific muscle and lead to variability in recruitment strategy between synergistic muscles, as noted in our study.

### 4.3 Matched relative intensity

In the present study, we sought to match the metabolic stress in NORM and HYPO at different absolute work‐rates. An alternative approach is to compare one‐ versus two‐legged cycling exercise which alters the metabolic stress at a constant work‐rate. Using this experimental design, EMG activity tended to rise faster during two‐legged cycling, which was interpreted as greater reliance on anaerobic metabolism due to a greater reserve in convective oxygen delivery in one‐legged cycling (Bundle et al., 2006). Thus, if the metabolic stress was normalized in HYPO, we might have expected similar EMG activity to NORM. However, we observed significantly lower muscle activation in HYPO, despite similar HR and ${\dot{V}}_{E}$. This result contrasts with Torres‐Peralta et al. (2014), who reported similar EMG activity when matched for relative intensity; however, a key difference in methodology is that step durations of only 2 min were used during incremental testing, whereas our protocol involved 4 min steps in the moderate domain and 8 min in the heavy domain. Hence, a step duration of only 2 min was likely insufficient to obtain a steady state metabolic response, which could have been exacerbated in the hypoxic condition. Our results indicate, however, that when a true metabolic steady state is achieved (which thereby requires sub‐RCP work rates), the relative metabolic stress does not appear to modulate the underlying muscle activation requirement of the task itself.

### 4.4 Task failure: Severe domain exercise

Studies involving small muscle mass tend to show progressively increasing EMG activity at high work‐rates, and this is exacerbated in HYPO, that is, the slope of the EMG versus time relationship is steeper (Fulco et al., 1996; Katayama et al., 2007). The present study is, however, the first to appropriately normalize the relative intensity during whole body exercise (two‐legged cycling) in both NORM and HYPO conditions to the RCP. We observed lower muscle activation at TF in HYPO compared to NORM; however, we also found lower muscle activation at TF in the constant‐load stage compared to PPO in both conditions regardless of the $F_{iO_{2}}$. An analysis of PO as a covariate was strongly significant, and therefore our results suggest that HYPO per se was not the primary factor influencing EMG activity at TF, but rather the workload requirement. This also may indicate that TF during large muscle mass exercise is not always associated with attainment of a similar muscle recruitment ceiling, but rather that muscle activation at TF can be constrained, likely due to inhibition of descending motor drive via group III/IV afferents (Hureau et al., 2016). Further evidence in favour of this has been shown by Rossman et al. (2014) who observed a greater rise in EMG activity during single‐leg knee extension as opposed to double‐leg knee extension. Hence this result, along with ours, aligns with the theory that muscle activation is not regulated to some critical upper limit, whereas task specificity plays a key role in underlying TF (Thomas et al., 2018).

### 4.5 Methodological considerations

The HYPO condition in this study corresponded to simulated altitude ∼3300 m, which is broadly classified as ‘moderate HYPO’ (Bartsch et al., 2004). This level was selected to elicit the largest decrease in oxygen transport while limiting the confounding effects of severe HYPO (≥4000 m), including excessive ventilatory drive, risk of acute mountain sickness, disproportionately large central fatigue (Amann et al., 2006; Goodall et al., 2010), and a systematic decrease in work capacity above critical power (Townsend et al., 2021). An increase in central fatigue with more severe simulated altitude has been suggested to result from a direct effect of cerebral HYPO on descending motor drive, as opposed to an indirect mechanism related to exacerbated peripheral fatigue and associated stimulation of group III/IV muscle afferents (Millet et al., 2012).

Another factor that could impact comparison to other studies is the choice of EMG normalization procedure. A common normalization method is to express the EMG activity as a percentage of the maximal isometric voluntary contraction (Hug & Dorel, 2009). However, this technique is only suitable for isometric tasks and does not reflect the MU recruitment strategy for dynamic muscle contraction. Therefore, we chose to normalize the rmsEMG to a percentage of the maximum voluntary dynamic effort in the unfatigued state, which has been used extensively and validated in numerous studies (Rouffet & Hautier, 2008).

### 4.6 Conclusion

The main finding of the present investigation is that moderate HYPO ($F_{iO_{2}}$ ≈ 0.135) did not alter quadriceps EMG activity at equivalent absolute intensities below the RCP, whereas it was lower when matched for relative intensity (i.e., equivalent metabolic stress). For exercise intensity above RCP, we also observed lower muscle activation at TF in HYPO compared to NORM; however, this was strongly associated with PO regardless of the work‐rate forcing protocol (ramp vs. CWR) or experimental condition (NORM vs. HYPO). These results do not support a role for moderate HYPO (below ∼3500 m equivalent altitude) in modulating MU recruitment and the firing rate control system during cycling exercise below the RCP. During ramp incremental exercise in HYPO, we found evidence of a decrease in EMG breakpoint, but this was not uniform amongst RF, VM and VL. Furthermore, PO at the EMG breakpoint was not strongly correlated to RCP for any muscle, in either NORM or HYPO. For exercise intensities in the severe domain >RCP, the results indicate that TF can occur at different levels of muscle activation and therefore attainment of a critical fatigue threshold is not associated with a fixed upper limit to muscle activation.

## AUTHOR CONTRIBUTIONS

Conception and experimental design: Dania Ibrahim, Youmna Elsayed Hassanein, and Nathan E. Townsend. Data collection: Dania Ibrahim, Youmna Elsayed Hassanein, and Nathan E. Townsend. Data analysis and interpretation: Dania Ibrahim, Youmna Elsayed Hassanein, and Nathan E. Townsend. Drafting of the work or revising it critically for important intellectual content: Dania Ibrahim and Nathan E. Townsend. All authors approved the final version of the manuscript and agree to be accountable for all aspects of the work in ensuring that questions related to the accuracy or integrity of any part of the work are appropriately investigated and resolved. All people designated as authors qualify for authorship, and all those who qualify for authorship are listed.

## CONFLICT OF INTEREST

None declared.

## Data Availability

The data that support the findings of this study are available from the corresponding author upon reasonable request.
